# Relationship between Serum Ferritin and Outcomes in β-Thalassemia: A Systematic Literature Review

**DOI:** 10.3390/jcm11154448

**Published:** 2022-07-30

**Authors:** Farrukh Shah, Krystal Huey, Sohan Deshpande, Monica Turner, Madhura Chitnis, Emma Schiller, Aylin Yucel, Luciana Moro Bueno, Esther Natalie Oliva

**Affiliations:** 1 Department of Haematology, Whittington Health NHS Foundation Trust, London N19 5NF, UK; 2 Bristol Myers Squibb, Princeton, NJ 08648, USA; krystal.a.huey@gmail.com (K.H.); aylin.yucel@bms.com (A.Y.); luciana.bueno@bms.com (L.M.B.); 3 Evidera, London W6 8BJ, UK; sohan.deshpande@evidera.com; 4 Evidera, Waltham, MA 02451, USA; monica.turner@evidera.com (M.T.); madhurakulkarni1082@gmail.com (M.C.); elenoxschill@gmail.com (E.S.); 5 Hematology Unit, Grande Ospedale Metropolitano Bianchi Melacrino Morelli, 89124 Reggio Calabria, Italy; enoliva@gmail.com

**Keywords:** β-thalassemia, iron overload, serum ferritin, systematic literature review

## Abstract

Among the difficulties of living with β-thalassemia, patients frequently require blood transfusions and experience iron overload. As serum ferritin (SF) provides an indication of potential iron overload, we conducted a systematic literature review (SLR) to assess whether SF levels are associated with clinical and economic burden and patient-reported outcomes (PROs). The SLR was conducted on 23 April 2020 and followed by analysis of the literature. Dual-screening was performed at the title, abstract, and full-text levels using predefined inclusion and exclusion criteria. Ten studies identified by the SLR were eligible for inclusion in the analysis. Seven studies were conducted in Europe, and most were prospective or retrospective in design. The patient populations had a median age of 20.7–42.6 years, with a percentage of men of 38–80%. Sparse data were found on the correlation between SF levels and mortality, and hepatic, skeletal, and cardiac complications; however, in general, higher SF levels were associated with worsened outcomes. The bulk of the evidence reported on the significant association between higher SF levels and endocrine dysfunction in its many presentations, including a 14-fold increase in the risk of diabetes for patients with persistently elevated SF levels. No studies reporting data on PROs or economic burden were identified by the SLR. SF levels provide another option for prognostic assessment to predict a range of clinical outcomes in patients with β-thalassemia.

## 1. Introduction

β-thalassemia is a group of hereditary blood disorders characterized by deviations in the synthesis of the β-chains of hemoglobin, which leads to an imbalance between α- and β-globin chains [1]. This imbalance can result in the early death of red blood cells and an array of conditions related to the inadequate production of red blood cells, commonly known as ineffective erythropoiesis. The phenotypic manifestations of ineffective erythropoiesis vary, ranging from clinically asymptomatic to severely anemic [2,3]. The management of anemia in this indication requires regular blood transfusions and increases intestinal iron absorption, which results in iron overload [4,5].

Iron chelation therapy (ICT) can decrease the morbidity associated with iron toxicity. Injectable deferoxamine is approved for long-term ICT in β-thalassemia and other iron overload diseases, and reduces hepatic iron levels as well as serum ferritin (SF) levels, which increases longevity. Its side effects include visual and auditory neurotoxicity and gastrointestinal effects. Oral chelator deferasirox has high affinity for iron but can cause renal failure in addition to gastrointestinal effects. Oral deferiprone is the chelator of choice for patients with inadequate responses to deferasirox and deferoxamine; the most frequent side effects include elevated liver enzymes, gastrointestinal disorders, and arthralgia [6]. SF acts as a buffer between iron deficiency and overload, and since the body cannot eliminate iron itself, even with treatment, it is an especially meaningful measure of iron levels [7].

The average levels of SF in healthy individuals range from 12 to 300 µg/L in men and from 12 to 150 µg/L in women [8]. SF levels >1000 µg/L are indicators of iron overload and are associated with harmful consequences, such as organ damage and increased mortality, a higher risk of cardiac events, and hepatic complications [9]. In a population of individuals with no endocrine or metabolic disorders, the prevalence of insulin resistance was higher for those with impaired SF levels (>300 µg/L for men and >200 µg/L for women) [10]. In a low-income urban population, there was a 0.5% increase in risk for every 10-unit rise in SF (pmol/L) for men, and a 5.1% increase for women [11].

Given these risks in healthy individuals, it is important to evaluate the effects of high SF levels in the context of β-thalassemia. A systematic literature review (SLR) was undertaken to explore the relationship between SF levels and outcomes of interest in patients with β-thalassemia, including clinical burden, patient-reported outcomes (PROs) (e.g., health-related quality of life and utility/disutility), and economic burden (healthcare resource utilization and costs).

## 2. Materials and Methods

A systematic search was conducted on 23 April 2020, and was followed by analysis of the literature according to well-established guidelines [12,13,14] to identify evidence on the clinical and economic burden and PROs for patients with β-thalassemia. Searches were developed based on guidelines such as the Preferred Reporting Items for Systematic Reviews and Meta-Analyses (PRISMA) [15] and the Cochrane Handbook for Systematic Reviews of Interventions [16]; electronic literature databases (i.e., Embase [via Ovid] and MEDLINE and MEDLINE In-Process [via Ovid]) were systematically searched to identify peer-reviewed studies reporting on the association between SF levels and the outcomes of interest in adults with β-thalassemia. Search strategies included both free-text and controlled vocabulary terms (Tables S1 and S2). 

Proceedings from recent meetings (January 2018 to April 2020) of the American Society of Hematology, the European Hematology Association, and the Professional Society for Health Economics and Outcomes Research (ISPOR) were searched for relevant abstracts. Researchers also examined the bibliographies of systematic reviews and/or meta-analyses identified during the electronic literature database searches that reported on β-thalassemia, and were published since January 2018, to identify additional relevant studies.

The titles and abstracts of relevant literature were evaluated during the first level of review using predefined inclusion and exclusion criteria, and the full text of articles from abstracts deemed relevant were retrieved and examined; criteria for inclusion and exclusion are provided in Table S3. Title, abstract, and full-text screenings were conducted by two independent investigators and any discrepancies were resolved by a third investigator.

None of the exclusion criteria and all protocol-specified inclusion criteria needed to be met for a study to pass the full-text level. Exclusion reasons were captured for references rejected at the full-text level and required agreement between both investigators. Studies for this SLR were required to assess the association between SF and the outcomes of interest in adults with β-thalassemia via a univariate or multivariate analytical approach. Extraction was performed by one researcher using an approved template and validated by a second researcher. Any discrepancies were resolved by a third senior researcher. The included studies were assessed for risk of bias using the Quality in Prognostic Studies (QUIPS) tool (https://methods.cochrane.org/prognosis/tools; accessed on 28 July 2022).

## 3. Results

**Figure 1:**
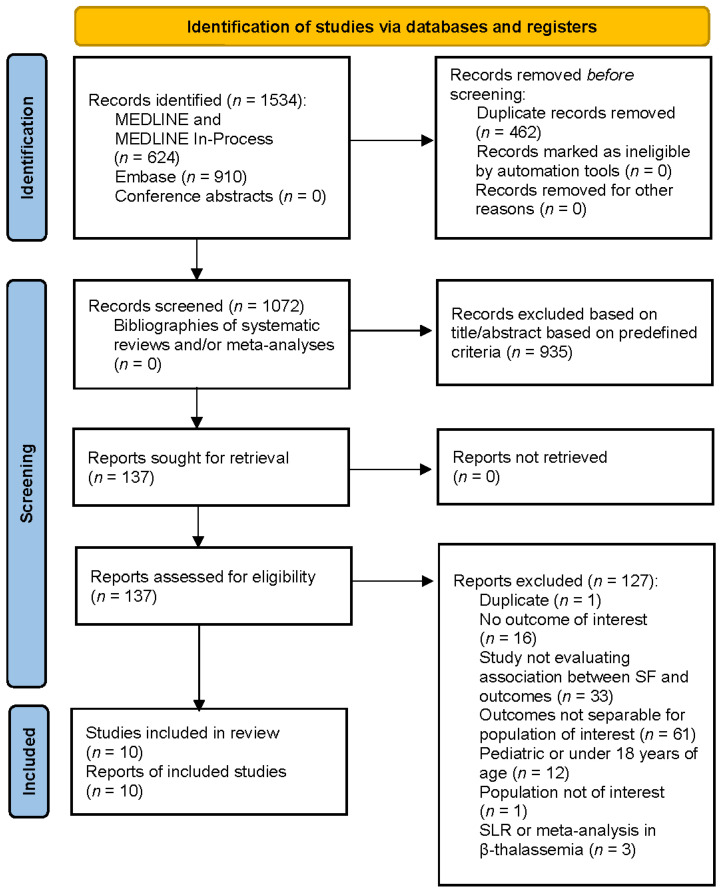
PRISMA diagram of attrition for β-thalassemia studies. SF, serum ferritin; SLR, systematic literature review.

A total of 1534 references were identified through the database searches. After removal of 462 duplicates, 1072 abstracts were screened and 137 progressed to full-text review; an additional 127 publications were excluded at this level, and reasons were documented. Supplementary searches of conference presentations did not identify any additional references, resulting in 10 full-text studies eligible for inclusion in the SLR [17,18,19,20,21,22,23,24,25,26]. The study attrition diagram is depicted in Figure 1.

Studies were assessed for quality using the QUIPS tool. Although most studies were rated with a moderate risk of bias (due to a lack of reporting on the variables for which the analyses controlled), those reporting on the β-thalassemia population generally showed a low risk of bias. One of the included studies [21] demonstrated a high risk of bias in its statistical analysis and presentation; reviewers determined that its presentation of results was poorly written, leading to potential confusion and an overall lack of clarity on the outcomes of interest. 

Five studies were prospective in design [19,20,21,23,26], four were retrospective [17,18,24,25], and one was cross-sectional [22]. Studies were primarily conducted in European countries (four in Italy [19,20,24,25], two in Greece [23,26], and one in the United Kingdom [UK] [17]), while two were conducted in Iran [21,22] and one in Taiwan [18]. Two of the Italian studies were linked, with each paper providing different outcome results for the same population [19,20]. Study characteristics are presented in Table 1.

The study populations included sample sizes from 27 to 165 patients with a mix of thalassemia type—major, intermedia, or both. The mean age ranged from 20.7 years (in a population with β-thalassemia intermedia without hydroxyurea) to 42.6 years (in a population with pulmonary hypertension), and the percentage of men ranged from 38% to 80%.

Half of the studies reported transfusion dependence and the type of treatments patients received, which was predominately deferoxamine. Deferasirox, deferiprone, and combination ICT therapy were also used [17,20,24,25,26].

SF levels at baseline were measured using a variety of units across the 10 studies, and these values ranged from 515 to 3149 µg/L, from 518 to 2094 ng/cc, and from 1293 to 1912 mg/g/dw in one study. Studies most often measured associations based on SF levels at baseline but also examined the average SF level over 10 years or the duration of the study period. Patient characteristics can be found in Table 2.

Univariate and multivariate models were used to report prognostic factors; four studies used univariate analyses, and six applied both. The statistical analyses used across studies included Cox proportional hazards, log rank test, Pearson correlation coefficient, Spearman correlation coefficient, Kaplan–Meier curves, linear regression, and Fisher’s exact tests; some studies used a combination of these analyses. 

The variables included in the multivariate models differed but were typically those that were deemed significant in previous univariate models; age and sex were frequently controlled for. Only four studies included either ICT or iron measures (liver and cardiac T2*) in their analyses [17,20,23,26].

### 3.1. Mortality

Higher SF levels were a significant predictor for mortality in a Greek population with β-thalassemia intermedia, reported in only one study [23]. As baseline SF levels increased, the risk of death significantly increased for each 1000 ng/mL increment in the univariate analysis (hazard ratio [HR]: 1.72, 95% confidence interval [CI], 1.30, 2.29, *p* < 0.0001) and the multivariate analysis (HR: 1.95, 95% CI, 1.22, 3.12, *p =* 0.005). All deaths occurred in patients with median SF ≥ 2800 ng/mL in the post-hoc analyses. The multivariate analysis controlled for sex, age at the start of deferoxamine treatment, SF concentrations before ICT, median SF concentrations, the proportion of ferritin measurements exceeding certain threshold values, and the degree of reduction in the SF concentrations approximately one and two years after the initiation of therapy (Table S4). 

### 3.2. Hepatic Complications

Of the ten studies, only one reported on hepatic complications; one study of Italian patients with β-thalassemia intermedia evaluated hepatic stiffness as a predictor of liver fibrosis (Table S5) [24]. Higher SF levels indicated greater hepatic stiffness, as transient elastography provided a measure of hepatic stiffness predictive of fibrosis. A linear regression analysis indicated that as SF levels increased, so did the rate of change in transient elastography values; this correlation was significant for the entire population (R^2^: 0.836, *p* < 0.001) as well as those patients who were non-chelated (R^2^: 0.806, *p* < 0.001) and chelated (R^2^: 0.758, *p* < 0.001). The study did not specify treatment details on the patients’ ICT.

### 3.3. Skeletal Complications

Two Iranian studies reported an association between increased SF levels at baseline and skeletal complications (Table S6) [21,22]. There was a significant risk of trabeculation (*p* = 0.028), rib widening (*p =* 0.015), and facial bone deformity (*p =* 0.009) associated with increasing ferritin levels following a univariate analysis in a population with β-thalassemia intermedia [22]. The second study included thalassemia major and intermedia populations and found that femoral bone mineral density (BMD) was significantly negatively correlated to SF among patients with osteopenia or osteoporosis (model coefficient − 0.52, *p* < 0.05); this trend did not hold for patients with normal BMD (model coefficient 0.12, *p* = not significant) [21]. A negative correlation was also observed for patients older than 20 years with osteopenia or osteoporosis (model coefficient − 0.561, *p* < 0.05, indicating lower bone density with higher SF), but no correlation for patients with normal BMD (model coefficient 0.239, *p =* not significant) [21]. Lumbar BMD was also significantly and negatively correlated to ferritin among patients older than 20 years with osteopenia or osteoporosis (model coefficient − 0.55, *p* < 0.05) and with normal BMD (model coefficient 0.466, *p* < 0.05) [21].

### 3.4. Cardiac Complications

Two studies analyzed cardiac complications and their association with SF levels (Table S7) [18,26]. In a Taiwanese study evaluating patients with β-thalassemia, the risk of deformation using a two-dimensional speckle tracking analysis identified longitudinal, circumferential, and radial strain. The risk of longitudinal strain was significantly correlated to an increasing ferritin level (r = 0.42, *p =* 0.012), whereas radial strain was significantly associated with a decreasing ferritin level (r = 0.41, *p =* 0.0163). There was no significant association between circumferential strain and ferritin levels (r = 0.17, *p =* 0.438). SF levels were lower, but not significantly so, in those without clinical events compared with the groups with cardiomegaly and clinical events (*p =* 0.06) [18].

In a thalassemia major population from Greece, there was a significant correlation between increased ferritin levels in the year prior to the study and tricuspid regurgitant jet velocity (a measure of pulmonary artery pressure). This was measured with a univariate analysis (R = 0.44, *p =* 0.019) and a multivariate analysis that controlled for age, ferritin level, and age at chelation onset (R = 0.48, *p =* 0.0328). Pulmonary hypertension could be predicted with a cutoff value for SF at 1350 μg/L in the multivariate analysis [26].

### 3.5. Endocrine Risk Factors

#### 3.5.1. Endocrine Disorders

Higher SF levels were linked to a higher risk of endocrinopathy, including thyroid and parathyroid dysfunction, diabetes mellitus, hypogonadism, osteoporosis, and renal and gallbladder lithiasis (Table S8). This risk was significant in an Italian study [20] of patients with thalassemia major and intermedia that used two multivariate models—one that adjusted for age and sex (β 0.26, *p =* 0.002) and another that adjusted for age, sex, and additional covariates known to be connected to endocrine dysfunction in patients with thalassemia (β 0.22, *p =* 0.01). There was also a significant decrease in the risk of multiple endocrinopathies when SF levels were <1800 μg/L (HR: 0.3, 95% CI, 0.1, 0.8; mean follow-up time to progression: 22 months). Both the univariate analysis (HR: 1.30, 95% CI, 1.18, 1.50; *p* < 0.0001) and the multivariate analysis (HR: 1.23, 95% CI, 1.13, 1.28; *p* < 0.0001) indicated a significantly increased risk of endocrine dysfunction (thyroid and parathyroid dysfunction, diabetes mellitus, hypogonadism, osteoporosis, and renal and gallbladder lithiasis) [20].

An SF level > 1300 ng/mL significantly predicted the development of a new endocrinopathy within five years in patients who did not have an existing endocrine disorder at baseline (area under the curve [AUC]: 0.810, *p =* 0.025) in a β-thalassemia major population from Italy. The best predictor for the reversal of endocrinopathy in those who had an existing endocrinopathy at baseline was an SF level of <200 ng/mL, although this did not reach statistical significance (AUC 0.746, *p =* 0.147) in the same population [25].

#### 3.5.2. Diabetes

The risk of diabetes mellitus was significantly higher for those with an average 10-year SF level >1500 μg/L (odds ratio [OR]: 3.4, 95% CI, 1.2, 9.6; *p =* 0.020) or >1250 μg/L (OR: 4.9, 95% CI, 1.3, 18.3; *p =* 0.016) compared with those with a lower average in a univariate analysis of a UK population with β-thalassemia major [17]. The multivariate analysis in the same study controlled for age, sex, and worst liver T2* and showed that average 10-year SF levels >1250 μg/L were significantly associated with diabetes mellitus (OR: 14.8, 95% CI, 2.4, 90.0; *p =* 0.003).

#### 3.5.3. Thyroid Function

Patients with thalassemia major and intermedia in an Italian study were more likely to progress to thyroid dysfunction (univariate analysis [HR: 1.36; 95% CI, 1.22, 1.59; *p* < 0.0001] and multivariate analysis [HR: 1.20, 95% CI, 1.10, 1.26; *p* < 0.0001]) and thyropathy (SF values >1800 mg/L [HR: 0.3, 95% CI, 0.1, 0.7], with a mean follow-up time of 14 months) [19]. Patients in the linked Italian study with ferritin values <1800 μg/L had a lower risk of developing thyroid dysfunction (HR: 0.3, 95% CI, 0.1, 0.7; *p =* 0.005) than those with SF >1800 μg/L; those with SF >1800 μg/L experienced significantly faster hypothyroidism development, with a mean follow-up time to progression of 14 months compared with > 40 months for those with lower levels [20]. However, a univariate analysis in a UK population with β-thalassemia major observed that the risk of hypoparathyroidism was not significantly associated with an average 10-year SF level of >1500 μg/L (OR: 0.4, 95% CI, 0.1, 1.3; *p =* 0.13) vs. a lower level [17].

#### 3.5.4. Hypogonadism

In an Italian population with β-thalassemia major and intermedia [19], the risk of hypogonadism was significantly associated with ferritin levels <893 μg/L (HR: 0.2, 95% CI, 0.1, 0.6) with a mean follow-up time to progression of 14 months. An average 10-year SF >2000 μg/L was also significantly associated with hypogonadism (OR: 2.9, 95% CI, 1.0, 8.3; *p =* 0.047) in a multivariate analysis in a UK population with β-thalassemia major [17]. However, it was not significantly associated with an average 10-year SF level of >2000 μg/L when compared with a lower average 10-year SF level in a univariate analysis (OR: 1.9, 95% CI, 0.8, 4.7; *p =* 0.18) [17].

## 4. Discussion

This SLR on the relationship between SF levels and outcomes of interest in patients with β-thalassemia identified sparse evidence that only provided insights on clinical outcomes. Generally, higher SF levels indicated worse outcomes in patients. The bulk of the SLR evidence reported on the significant association between higher SF levels and endocrine dysfunction in its many presentations. The most striking example was the 14-fold increase in the risk of diabetes for patients with persistently elevated SF levels, highlighting the additional burden that can develop in patients with β-thalassemia if SF levels remain too elevated for too long. Evidence also indicated that patients had increased the frequency of complications in target organs such as the heart and liver. Patients also experienced a heightened likelihood of mortality, with the risk of death increasing as SF levels rose. Given that β-thalassemia is a lifelong illness, the burden is experienced by patients during multiple phases of life.

Although SF levels were demonstrated to be prognostic indicators in β-thalassemia, the variation in SF thresholds and how “higher” SF levels are defined highlight the difficulty in its broad application in similar outcomes. Only one study focused on the association between SF and mortality in the SLR, likely due to the lifelong nature of β-thalassemia and the practical difficulties in following patients’ in the long term or until death. Likewise, other outcomes that were evaluated in the identified studies, such as the confirmation of organ and skeletal damage, may develop after many years; this limits the ability to accurately assess the correlation between higher SF levels and worsened outcomes. To improve evidence in this area, more studies are needed that provide data on long-term follow-up, so that clinical outcomes may be monitored and analyzed over time. Furthermore, very few outcomes had findings confirmed across both univariate and multivariate analyses, complicating the assessment of outcomes with multiple potential influential factors.

The SLR had no limitations on countries of interest for inclusion, and five countries were discussed across the included literature. The majority of the evidence was identified from studies conducted in Greece, Iran, and Italy; without details on treatments prescribed, however, no conclusions could be drawn on differences in treatment patterns or healthcare systems across countries. The degree to which such differences could potentially impact outcomes was therefore unclear, though the question would be valuable for further exploration. In addition, no data on the impact of SF on PROs or economic burden were identified by the SLR, although worsened outcomes would be expected to negatively impact both. Additional research study is needed to address this data gap.

Given the fact that high SF levels are treated with iron chelation, the burden of ICT in patients with β-thalassemia was assessed in a parallel review of SLRs, but no relevant literature was identified. Targeted searches were conducted in this population to identify details on clinical, economic, and humanistic burdens. Studies indicated that ICT did not provide substantial improvements in quality of life, but that the economic burden of long-term treatment with ICT was high. Patients also continued to experience endocrine dysfunction despite lifelong treatment with ICT [17,19,20,25,27,28,29,30,31,32,33,34,35,36,37,38,39,40,41,42,43,44,45,46,47,48,49].

This analysis did not provide conclusive data on the impact of SF on PROs or economic burden, and only a few studies were available for certain outcomes. The observed variation in SF thresholds used and the overall short time of follow-up constitute additional limitations. In summary, this SLR was conducted with a high degree of rigor, providing a reproducible study design and limiting the opportunity for bias, but the lack of available evidence limits the broader interpretation of the findings.

## 5. Conclusions

SF levels provide another option for prognostic factors to predict a range of clinical outcomes in patients with β-thalassemia. Additional research into the relationship between SF levels and economic or humanistic burdens will provide valuable insight into the full experience of patients with β-thalassemia.

## Figures and Tables

**Table 1:** Study characteristics of β-thalassemia studies.

Author, Year	Study Design	Country	Setting	Duration of Follow-Up	Sample Size (*n*)	Outcomes of Interest Reported
Ang, 2014 [17]	Retrospective cohort	UK	Hospital	NR	92	Endocrine complications
Chen, 2015 [18]	Retrospective case-control	Taiwan	NR	Median: 2.8 years	37	Cardiac outcomes
Chirico, 2013 [19]	Prospective cohort with randomized phase	Italy	Outpatient or clinic	NR	Total: 72 TM: 51 TI: 21	Endocrine complications
Chirico, 2015 [20]	Prospective cohort	Italy	Outpatient or clinic	NR	Overall: 72 TM: 51 TI: 21	Endocrine complications
Ebrahimpour, 2012 [21]	Prospective cross-sectional	Iran	Outpatient or clinic	NR	Overall: 80 Subgroup with normal BMD: 50 Subgroup with osteomalacia/osteoporosis: 30	Skeletal outcomes
Foroughi, 2015 [22]	Cross-sectional	Iran	Outpatient or clinic	NR	Total: 86 TM: 39 TI: 47	Skeletal outcomes
Hahalis, 2009 [23]	Prospective case-control	Greece	Outpatient or clinic	Mean: 10.7 years	36	Mortality
Musallam, 2012 [24]	Retrospective cohort	Italy	Outpatient or clinic	4 years	Overall: 42 Non-chelated: 28 Chelated: 14	Liver outcomes
Poggi, 2016 [25]	Retrospective cohort	Italy	Outpatient or clinic	NR	165	Endocrine complications
Vlahos, 2012 [26]	Prospective cohort	Greece	Hospital	NR	27	Pulmonary outcomes

Abbreviations: BMD, bone mineral density; NR, not reported; TI, thalassemia intermedia; TM, thalassemia major; UK, United Kingdom.

**Table 2:** Patient characteristics of β-thalassemia studies.

Age	Male Sex	Time Since Diagnosis	TM Patients	TI Patients	Definition of TD/ TD Patients	Patients Receiving Transfusion	Baseline Serum Ferritin
Chirico, 2013 [19]; Italy							
TM, mean (SD): 34.4 (9.7) years TI, mean (SD): 38.5 (19.4) years	Overall: 47.2%	NR	70.8%	29.2%	Definition NR (TM patients presumed TD) TM: 100% TI: 48%	TM: 100% TI: 76%	TM, mean (range): 872 (541–1921) µg/L TI, mean (range): 670 (480–1345) µg/L
Foroughi, 2015 [22]; Iran							
β-TI with hydroxyurea, mean (SD): 26.7 (7.8) years β-TI without hydroxyurea, mean (SD): 20.7 (7.2) years	β-TI with hydroxyurea: 64% β-TI without hydroxyurea: 50%	NR	0	100%	NR NR	NR	β-TI with hydroxyurea, mean (SD): 762 (602) ng/cc β-TI without hydroxyurea, mean (SD): 518 (461) ng/cc
Ang, 2014 [17]; UK							
Mean (range): 36 (18–59) years	48%	NR	100%	0	Definition NR (TM patients presumed TD) 100%	100%	Median 10-year average (range): 2042 (501–10,101) µg/L
Chen, 2015 [18]; Taiwan							
Mean (SD): 24.2 (5.5) years	56.8%	Mean (SD): 22.04 (6.5) years (time from disease onset)	NR	NR	NA NR	100%	Mean (SD): 2476.8 (300.6)
Chirico, 2015 [20]; Italy							
TM, mean (SD): 34.4 (9.7) years TI, mean (SD): 38.5 (19.4) years	TM: 51% TI: 38%	NR	Of overall: 70.8%	Of overall: 29.2%	Definition NR For TI: 48% dependent on hemotransfusion, 28% occasionally transfused	Of overall: 93%	TM, mean (range): 872 (541–1921) µg/L TI, mean (range): 670 (480–1345) µg/L
Ebrahimpour, 2012 [21]; Iran							
Normal BMD, mean (SD): 24.55 (0.75) years Osteomalacia/ osteoporosis, mean (SD): 28.69 (1.56) years	Normal BMD: 57% Osteomalacia/ osteoporosis: 52%	NR	61%	39%	Regular transfusion therapy: 100%	100%	Normal BMD, mean (SD): 1912.21 (247.95) mg/g/dw Osteomalacia/ osteoporosis, mean (SD): 1293.39 (249.76) mg/g/dw
Hahalis, 2009 [23]; Greece							
At study start, mean (SD): 23 (5) years At study termination, mean (SD): 37 (6) years At time of death, mean (SD): 25 (3) years	44%	NR	100%	0	Need for regular transfusions since the first months of life: 100%	100%	Median (IQR), ng/mL: 3140 (2150–3880)
Musallam, 2012 [24]; Italy							
Overall, median (range): 38 (26–54) years	50%	NR	0	100%	NR 0	NR	Overall: first measurement, µg/L; median (IQR): 580.0 (332.3–925.3) Non-chelated: first measurement, µg/L; median (IQR): 515.0 (302.5–901.3) Chelated: first measurement, µg/L; median (IQR): 636.5 (415.5–1554.5)
Poggi, 2016 [25]; Italy							
Mean (SD): 39.9 (8.3) years	43%	NR	100%	0	Definition NR (TM): 100%	100%	Median (range) over 5 years: 555 ng/mL (63–6140)
Vlahos, 2012 [26]; Greece							
Pulmonary HTN absent, mean (SD): 37.7 (7.7) years Pulmonary HTN present, mean (SD): 42.6 (14.5) years	Pulmonary HTN absent: 68.2% Pulmonary HTN present: 80%	NR	100%	0	Definition NR (TM patients): 100%	100%	Pulmonary HTN absent, mean (SD): 1181 (755) µg/L Pulmonary HTN present, mean (SD): 2440 (1836) µg/L

Abbreviations: BMD, bone mineral density; HTN, hypertension; IQR, interquartile range; NR, not reported; SD, standard deviation; TD, transfusion dependent; TI, thalassemia intermedia; TM, thalassemia major; UK, United Kingdom.

## Data Availability

The data that support the findings of this study are available in the supplementary material of this article and from the corresponding author upon reasonable request.

## Supplementary Materials

The following are available online at https://www.mdpi.com/article/10.3390/jcm11154448/s1, Table S1: Medline (via Ovid) search strategy for beta-thalassemia population, Table S2: Embase (via Ovid) search strategy for beta-thalassemia population, Table S3: Screening/eligibility criteria for β-thalassemia, Table S4: Serum ferritin and survival in β-thalassemia studies, Table S5: Serum ferritin and liver fibrosis in β-thalassemia studies, Table S6: Serum ferritin and skeletal outcomes in β-thalassemia studies, Table S7: Serum ferritin and cardiac or pulmonary outcomes in β-thalassemia studies, Table S8: Serum ferritin and endocrine complications in β-thalassemia studies, Table S9: QUIPS quality assessment of β-thalassemia studies.
