# Supplementary material for: Relationship between Serum Ferritin and Outcomes in β-Thalassemia: A Systematic Literature Review
Source: J Clin Med. 2022 Jul 30;11(15):4448. doi: 10.3390/jcm11154448 (PMC9369259; doi:10.3390/jcm11154448)
Supplement: Supplementary file 1 [file jcm-11-04448-s001.zip › jcm-1793355-supplementary.pdf]

Supplemental Material

SUPPLEMENTARY TABLE S1. Medline (via Ovid) search strategy for beta-thalassemia population.

Search number	Search string
1.	exp beta-Thalassemia/ or beta-thalassemia.tw. or thalassemia.tw. or thalassaemia.tw. or (cooley* or cooleys) and (anemia or anaemia)).tw.
2.	exp Iron-Binding Proteins/ or serum ferritin.mp.
3.	1 and 2
4.	(animals not humans).sh.
5.	(case report or case series or woman or man or child or adolescent or female or male or boy or girl or infant).ti.
6.	case reports/ or case report$.jw.
7.	(Ephemera or Introductory Journal Article or News or Newspaper Article or Editorial or Comment or Overall or Letter or Congress).pt. or In Vitro Techniques/ or (commentary or editorial or comment or letter or congress or mice or rat or mouse or animal or murine).ti.
8	review.pt. not (systematic or meta$).mp.
9.	3 not (4 or 5 or 6 or 7 or 8)
10.	(pediatric$ or paediatric$ or preterm$ or newborn$ or child$ or infant$ or infancy or neonat$ or preschool$ or young$ or early years or adolescen$ or teenage$ or preteen$ or youth or girl$ or boy$ or student$ or juvenile$ or minor or minors or baby or babies).ti.
11.	(pediatric$ or paediatric$ or preterm$ or newborn$ or child$ or infant$ or infancy or neonat$ or preschool$ or young$ or early years or adolescen$ or teenage$ or preteen$ or youth or girl$ or boy$ or student$ or juvenile$ or minor or minors or baby or babies).ti. and (exp adult/ or adult$.ti.)
12.	10 not 11
13.	9 not 12
14.	limit 13 to english language

15.	limit 13 to yr="2009 -Current"

SUPPLEMENTARY TABLE S2. Embase (via Ovid) search strategy for beta-thalassemia population.

Search number	Search string
1.	exp beta thalassemia/ or beta-thalassemia.tw. or beta thalassemia.tw. or thalassemia.tw. or thalassaemia.tw. or ((cooley* or cooleys) and (anemia or anaemia)).tw.
2.	exp ferritin blood level/ or serum ferritin.mp.
3.	1 and 2
4.	limit 3 to (article or article in press)
5.	(animal not human).sh.
6.	(case report or case series or woman or man or child or adolescent or female or male or boy or girl or infant).ti.
7.	case study/ or case report$.jx. or case report$.jw.
8	(editorial or note or letter).pt. or in vitro Techniques/ or in vitro study/ or (commentary or editorial or comment or letter or mice or rat or mouse or animal or murine).ti.
9.	review.pt. not (systematic or meta$).mp.
10.	4 not (5 or 6 or 7 or 8 or 9)
11.	(pediatric$ or paediatric$ or preterm$ or newborn$ or child$ or infant$ or infancy or neonat$ or preschool$ or young$ or early years or adolescen$ or teenage$ or preteen$ or youth or girl$ or boy$ or student$ or juvenile$ or minor or minors or baby or babies).ti.
12.	(pediatric$ or paediatric$ or preterm$ or newborn$ or child$ or infant$ or infancy or neonat$ or preschool$ or young$ or early years or adolescen$ or teenage$ or preteen$ or youth or girl$ or boy$ or student$ or juvenile$ or minor or minors or baby or babies).ti. and (exp adult/ or adult$.ti.)
13.	11 not 12
14.	10 not 13
15.	limit 14 to english language
16.	Limit 11 to yr="2009 -Current"

SUPPLEMENTARY TABLE S3. Screening/eligibility criteria for β-thalassemia.

Domain	Inclusion criteria	Exclusion criteria
Population	Adults (≥18 years) with β-thalassemia	Publications reporting on children or healthy volunteers
Prognostic/ predictive factors	Studies must have assessed and reported SF levels using quantitative methods.[a]	NA
Outcomes	**Clinical outcomes**<br>• Incidence of complications related to iron overload, including cardiac failure, hypogonadism, hypothyroidism, carcinoma, diabetes, liver failure<br>• Progression to high-risk disease<br>• Overall survival<br>• Treatment duration<br>• Subsequent therapies, or combinations of different types of ICTs, or maintenance on personalized regimen<br>• Total mortality<br>• Liver fibrosis, stiffness, or siderosis<br>• Skeletal outcomes such as bone disease, density, osteoporosis, skeletal changes, or fracture<br>• Cardiac siderosis<br>• Pulmonary hypertension<br>• Fertility<br>**Humanistic outcomes:**<br>• Utility studies	Publications that only report data on pharmacokinetics/pharmacodynamics

Domain	Inclusion criteria	Exclusion criteria
	• Health-related quality of life (e.g., EQ-5D, SF-36, and EORTC QLQ-C30)<br>**Economic outcomes:**<br>• Healthcare resource utilization<br>Specialist visits<br>Unscheduled physician visits<br>Emergency room visits<br>Transfusion clinic visits<br>Hospitalization<br>• Costs<br>Direct costs<br>Total treatment costs<br>Costs of healthcare and social care<br>Indirect costs<br>Productivity<br>Absenteeism and presenteeism	
Study designs	• Observational cohort studies (prospective or retrospective)<br>• RCTs	Publications of studies with the following designs:<br>• Animal studies<br>• *In vitro/ex vivo* studies<br>• Gene expression/protein expression studies<br>• Narrative publications<br>• Non-systematic reviews<br>• Case studies<br>• Case reports

Domain	Inclusion criteria	Exclusion criteria
		• Editorials<br>• Systematic reviews and meta-analysis (Will be included for reference checking only. Full papers will be excluded using a separate exclusion code.)
Duplicate	If duplicates are identified, the copy of the record with the lower refID number will be included	Publications that are duplicates of other publications in the search yield<br>The copy of the record with higher refID number will be excluded.
Study limits	Only English language articles/conference abstracts will be included<br>• Studies published from 2009 to present[b]<br>• Conference proceedings from 2018 to present will be searched	Journal articles and conference abstracts without English full text<br>Studies published outside the timeframe of interest
Geography	None	

Abbreviations: EORTC QLQ-C30, European Organization for Research and Treatment of Cancer Quality of Life Questionnaire Version 3.0; EQ-5D, European Quality of Life Five Dimension; ICT, iron chelation therapy; RCT, randomized controlled trial; SF-36, 36-item Short Form Survey; SF, serum ferritin.

[a]Studies assessing efficacy of an intervention without investigating the relationship of SF and the outcomes were excluded. Studies assessing the relationship between SF level and any of the outcomes of interest listed in the table above were of interest. Studies with quantitative outcomes refer to those based on univariate or multivariate models or adjusted analysis with some kind of quantification results, as opposed to studies making unsupported statements about association in the discussion (which were excluded).

[b]This limit ensured the most recent and relevant data.

SUPPLEMENTARY TABLE S4. Serum ferritin and survival in β-thalassemia studies.

**Study author, year country study design**	**Population (n)**	**Univariate or multivariable and type of statistical analysis performed**	**Model variables**	**When SF evaluated**	**Continuous or categorical, and categories**	**Outcome**	**Effect**
Hahalis, 2009 [1] Greece Prospective case-control	Overall 36	Univariate Cox proportional hazards	None	Baseline	Categorical Per 1000 ng/mL SF	Mortality	HR (95% CI), 1.72 (1.3, 2.29) *P* < 0.0001
	Overall 36	Multivariable Cox proportional hazards	Variables included sex, age at the start of deferoxamine treatment, SF concentrations before chelation therapy, median SF concentrations, proportion of ferritin measurements exceeding certain threshold values, and degree of reduction in the SF concentrations approximately 1 and 2 years after initiation of therapy	Baseline	Categorical Per 1000 ng/mL SF	Mortality	HR (95% CI), 1.95 91.22, 3.12) *P* = 0.005

Abbreviations: CI, confidence interval; HR, hazard ratio; SF, serum ferritin.

SUPPLEMENTARY TABLE S5. Serum ferritin and liver fibrosis in β-thalassemia studies.

**Study author, year country study design**	**Population (n)**	**Univariate or multivariable and type of statistical analysis performed**	**Model variables**	**When serum SF**	**Continuous or categorical, and categories**	**Outcome**	**Effect**
Musallam, 2012[2]<br>Italy<br>Retrospective cohort	Overall<br>42	Univariate<br>Linear regression	None	Throughout study period	Continuous	Liver fibrosis	Model coefficient: R^2: 0.836<br>*P* < 0.001
	Subgroup: Non-chelated group: 28	Univariate<br>Linear regression	None	Throughout study period	Continuous	Liver fibrosis	Model coefficient: R^2: 0.806<br>*P* < 0.001
	Subgroup: Chelated group: 14	Univariate<br>Linear regression	None	Throughout study period	Continuous	Liver fibrosis	Model coefficient: R^2: 0.758<br>*P* < 0.001

Abbreviation: SF, serum ferritin.

SUPPLEMENTARY TABLE S6. Serum ferritin and skeletal outcomes in β-thalassemia studies.

Study author, year country study design	Population (n)	Univariate or multivariable and type of statistical analysis performed	Model variables	When serum ferritin evaluated	Continuous or categorical, and categories	Outcome	Effect
Foroughi, 2015[3] Iran Cross-sectional	Subgroup: β-thalassemia intermedia patients 47	Univariate T test or chi square	Sex, age, hemoglobin, RBC, platelet, NRBC and ferritin level	Baseline	Continuous	Skeletal complications: trabeculation	*P* = 0.028
		Univariate T test or chi square	Gender, age, hemoglobin, RBC, platelet, NRBC and ferritin level	Baseline	Continuous	Skeletal complications: rib widening	*P* = 0.015
		Univariate T test or chi square	Gender, age, hemoglobin, RBC, platelet, NRBC and ferritin level	Baseline	Continuous	Skeletal complications: facial bone deformity	*P* = 0.009

Ebrahimpour, 2012[4]<br>Iran<br>Prospective cross-sectional	Subgroup: Patients ≥20 years old with osteomalacia/osteoporosis<br>NR	Univariate T test	None	Baseline	Continuous	BMD	Model coefficient: -0.561, *P* < 0.05
	Subgroup: Patients ≥20 years old with normal BMD<br>NR	Univariate T test	None	Baseline	Continuous	BMD	Model coefficient: 0.239
	Subgroup: Patients ≥20 years old with osteomalacia/ osteoporosis<br>NR	Univariate T test	None	Baseline	Continuous	BMD	Model coefficient:-0.55, *P* < 0.05
	Subgroup: Patients ≥20 years old with normal BMD<br>NR	Univariate T test	None	Baseline	Continuous	BMD	Model coefficient: 0.466, *P*<0.05
	Subgroup: Patients with osteomalacia/ osteoporosis<br>30	Univariate T test	None	Baseline	Continuous	BMD	Model coefficient: -0.52, *P* < 0.05
	Subgroup: Patients with normal BMD<br>50	Univariate T test	None	Baseline	Continuous	BMD	Model coefficient: 0.12

Abbreviations: BMD, bone mineral density; NR, not reported; NRBC, nucleated red blood cell; RBC, red blood cell.

SUPPLEMENTARY TABLE S7. Serum ferritin and cardiac or pulmonary outcomes in β-thalassemia studies.

Study author, year country study design	Population (n)	Univariate or multivariable and type of statistical analysis performed	Model variables	When SF evaluated	Continuous or categorical, and categories	Outcome	Effect
Chen, 2015[5] Taiwan Retrospective Case-control	Subgroup: All β-thalassemia in study: 37	Univariate Pearson or Spearman correlation coefficients	NR	Baseline	Continuous	Cardiac failure: longitudinal strain	Model coefficient: r = 0.42 *P* = 0.012
	Subgroup: All β-thalassemia in study: 37	Univariate Pearson or Spearman correlation coefficients	NR	Baseline	Continuous	Cardiac failure: radial strain	Model coefficient: r = 0.41 *P* = 0.0163
	Subgroup: All β-thalassemia in study: 37	Univariate Pearson or Spearman correlation coefficients	NR	Baseline	Continuous	Cardiac failure: circumferential strain	Model coefficient: r = 0.17 *P* = 0.438
	Subgroup: All β-thalassemia in study: 37	Multivariable Cox proportional hazards	Age, sex, renal function, SF level, and echocardiographic covariates including LV	Baseline	Continuous	Cardiac events or death	*P* = 0.06

			mass index and ejection fraction				
Vlahos, 2012 [6] Greece Prospective cohort	Overall: 27	Univariate Logistic regression	None	The average value from 10 consecutive values taken during a period of 12 ± 13 months prior to the echocardiographic assessment	Continuous	Pulmonary hypertension	Model coefficient: R = 0.44 *P* = 0.019
	Overall: 27	Multivariable Logistic regression	Age, ferritin level and age at chelation onset	The average value from 10 consecutive values taken during a period of 12 ± 13 months prior to the echocardiographic assessment	Continuous	Pulmonary hypertension	Model coefficient: R = 0.48 *P* = 0.0328

Abbreviations: LV, left ventricular; NR, not reported; SF, serum ferritin.

SUPPLEMENTARY TABLE S8. Serum ferritin and endocrine complications in β-thalassemia studies.

Study author, year country study design	Population (n)	Univariate or multivariable and type of statistical analysis performed	Model variables	When serum ferritin evaluated	Continuous or categorical, and categories	Outcome	Effect
Chirico, 2013 [7] Italy Prospective cohort with randomized phase	Overall 72	Multivariable Cox proportional hazards	NR	Baseline	Continuous	Incidence of thyroid dysfunction	HR (95% CI), 1.2 (1.10, 1.26) *P* < 0.0001
	Overall 72	Univariate Pearson or Spearman correlation coefficients	NR	Baseline	Continuous	Incidence of thyroid dysfunction	HR (95% CI), 1.36 (1.22, 1.59) *P* < 0.0001
	Overall 72	Univariate Log rank test	NR	Baseline	Categorical SF >1800 µg/L vs. SF <1800 µg/L	Time to thyropathy	HR (95% CI), 0.3 (0.1, 0.7)
Ang, 2014[8] UK Retrospective cohort	Overall 92	Univariate Logistic regression	Age, worst liver T2* and average SF	Average over 10 years	Categorical Average 10-year SF of >1500 µg/L	Hypogonadism	OR (95% CI), 1.9 (0.8, 4.7) *P* = 0.18
	Overall 92	Univariate Logistic regression	Age, worst liver T2* and average SF	Average over 10 years	Categorical Average 10-year SF of >1500 µg/L	Hypoparathyroidism	OR (95% CI), 0.4 (0.1, 1.3) *P* = 0.13

**Study author, year country study design**	**Population (n)**	**Univariate or multivariable and type of statistical analysis performed**	**Model variables**	**When serum ferritin evaluated**	**Continuous or categorical, and categories**	**Outcome**	**Effect**
	Overall 92	Multivariable Logistic regression	Age, worst liver T2* and average SF	Average over 10 years	Categorical Average 10-year SF of >2000 µg/L	Hypogonadism	OR (95% CI), 2.9 (1.0, 8.3) *P* = 0.047
	Overall 92	Univariate Logistic regression	Age, worst liver T2* and average SF	Average over 10 years	Categorical Average 10-year SF of >1500 µg/L	Diabetes mellitus	OR (95% CI), 3.4 (1.2, 9.6) *P* = 0.02
	Overall 92	Univariate Logistic regression	Age, worst liver T2* and average SF	Average over 10 years	Categorical Average 10-year SF of >1250 µg/L	Diabetes mellitus	OR (95% CI), 4.9 (1.3, 18.3) *P* = 0.016
	Overall 92	Multivariable Logistic regression	Age, worst liver T2* and average SF	Average over 10 years	Categorical Average 10-year SF of >1250 µg/L	Diabetes mellitus	OR (95% CI), 14.8 (2.4, 90.0) *P* = 0.003
Chirico, 2015[9] Italy Prospective cohort	Overall 72	Multivariable Logistic regression	Age, sex	Baseline	Continuous	Endocrine complications	Model coefficient: β: 0.26 *P* = 0.002
	Overall 72	Multivariable Logistic regression	Age, sex, and other covariates that are causally linked to	Baseline	Continuous	Endocrine complications	Model coefficient: β: 0.22

Study author, year country study design	Population (n)	Univariate or multivariable and type of statistical analysis performed	Model variables	When serum ferritin evaluated	Continuous or categorical, and categories	Outcome	Effect
			endocrine diseases in thalassemic patients				P = 0.01
	Overall 72	Univariate Kaplan–Meier curves	None	Baseline	Categorical SF >1800 µg/L vs. SF <1800 µg/L	Hypothyroidism	HR (95% CI), 0.3 (0.1, 0.7) P = 0.005
	Overall 72	Univariate Kaplan–Meier curves	None	Baseline	Categorical SF >893 µg/L vs. SF <893 µg/L	Hypogonadism	HR (95% CI), 0.2 (0.1, 0.6) P = 0.001
	Overall 72	Univariate Kaplan–Meier curves	None	Baseline	Categorical SF >2300 µg/L vs. SF <2300 µg/L	Endocrine complications	HR (95% CI), 0.3 (0.1, 0.8) P = 0.02
	Overall 72	Univariate Cox proportional hazards	None	Baseline	Continuous	Endocrine complications	HR (95% CI), 1.3 (1.18, 1.50) P < 0.0001
	Overall 72	Multivariable Cox proportional hazards	Age, markers of iron overload (SF, liver, and cardiac*T2 values), hemoglobin	Baseline	Continuous	Endocrine complications	HR (95% CI), 1.23 (1.13, 1.28) P < 0.0001

Study author, year country study design	Population (n)	Univariate or multivariable and type of statistical analysis performed	Model variables	When serum ferritin evaluated	Continuous or categorical, and categories	Outcome	Effect
			levels, splenectomy, and HCV infection				
Poggi, 2016[10] Italy Retrospective cohort	Overall 165	Univariate Chi-squared and Fisher exact tests	None	Over the study period of 5 years	Categorical SF level >1300 ng/mL over 5 years	Endocrine complications	Model coefficient: AUC: 0.810 P = 0.025
	Overall 165	Univariate Chi-squared and Fisher exact tests	None	Over the study period of 5 years	Categorical SF level <200 ng/mL at baseline	Endocrine complications	Model coefficient: AUC: 0.746 P = 0.147

Abbreviations: AUC, area under the curve; CI, confidence interval; HCV, hepatitis C virus; HR, hazard ratio; NR, not reported; OR, odds ratio; SF, serum ferritin; UK, United Kingdom.

SUPPLEMENTARY TABLE S9. QUIPS quality assessment of β-thalassemia studies.

**Author and year of publication**	**Summary study participation**	**Study attrition summary**	**PF measurement summary**	**Outcome measurement summary**	**Study confounding summary**	**Statistical analysis and presentation summary**
	The study sample represents the population of interest on key characteristics, sufficient to limit potential bias of the observed relationship between PF and outcome.	Loss to follow-up (from baseline sample to study population analyzed) is not associated with key characteristics (i.e., the study data adequately represent the sample) sufficient to limit potential bias to the observed relationship between PF and outcome.	*PF* is adequately measured in study participants to sufficiently limit potential bias.	*Outcome of interest* is adequately measured in study participants to sufficiently limit potential bias.	Important potential confounders are appropriately accounted for, limiting potential bias with respect to the relationship between *PF* and *outcome*.	The statistical analysis is appropriate for the design of the study, limiting potential for presentation of invalid or spurious results.
Chirico, 2013	Low risk	Low risk	Low risk	Low risk	Moderate risk	Low risk
Foroughi, 2015	Low risk	Low risk	Low risk	Low risk	Moderate risk	Low risk
Ang, 2013	Low risk	Low risk	Low risk	Low risk	Moderate risk	Low risk
Chen, 2015	Low risk	Low risk	Low risk	Low risk	Moderate risk	Low risk
Chirico, 2015	Low risk	Low risk	Low risk	Low risk	Low risk	Low risk

**Author and year of publication**	**Summary study participation**	**Study attrition summary**	**PF measurement summary**	**Outcome measurement summary**	**Study confounding summary**	**Statistical analysis and presentation summary**
Ebrahimpour, 2012	Low risk	Low risk	Low risk	Low risk	Moderate risk	High risk
Hahalis, 2009	Low risk	Low risk	Low risk	Low risk	Low risk	Low risk
Musallam, 2012	Low risk	Low risk	Low risk	Low risk	Moderate risk	Low risk
Poggi, 2016	Moderate risk	Low risk	Low risk	Low risk	Moderate risk	Low risk
Vlahos, 2012	Moderate risk	Low risk	Low risk	Low risk	Moderate risk	Low risk

Abbreviations: PF, prognostic factor; QUIPS, Quality in Prognostic Studies; SF, serum ferritin.

SUPPLEMENTARY REFERENCES

1. Hahalis G, Kourakli A, Gerasimidou I, Kalogeropoulos AP, Sitafidis G, Papageorgiou U, et al. Cardiac mortality in {beta}-thalassemia major: resting but not dobutamine stress echocardiography predicts mortality among initially cardiac disease-free patients in a prospective 12-year study. *Eur J Heart Fail.* 2009; **11**(12): 1178–81.
2. Musallam KM, Motta I, Salvatori M, Fraquelli M, Marcon A, Taher AT, et al. Longitudinal changes in serum ferritin levels correlate with measures of hepatic stiffness in transfusion-independent patients with beta-thalassemia intermedia. *Blood Cells Mol Dis*. 2012; **49**(3–4): 136–9.
3. Foroughi AA, Ghaffari H, Haghpanah S, Nazeri M, Ghaffari R, Bardestani M, et al. Comparative study of radiographic and laboratory findings between beta thalassemia major and beta thalassemia intermedia patients with and without treatment by hydroxyurea. *Iran Red Crescent Med J*. 2015; **17**:(2): e23607.
4. Ebrahimpour L, Akhlaghpoor S, Azarkayvan A, Salehi M, Morteza A, Alinaghi R. Correlation between bone mineral densitometry and liver/heart iron overload evaluated by quantitative T2 MRI. *Hematology*. 2012; **17**(5): 297–301.
5. Chen MR, Ko HS, Chao TF, Liu HC, Kuo JY, Bulwer BE, et al. Relation of myocardial systolic mechanics to serum ferritin level as a prognosticator in thalassemia patients undergoing repeated transfusion. *Echocardiography.* 2015; **32**(1): 79–88.
6. Vlahos AP, Koutsouka FP, Papamichael ND, Makis A, Baltogiannis GG, Athanasiou E, et al. Determinants of pulmonary hypertension in patients with beta-thalassemia major and normal ventricular function. *Acta Haematol*. 2012; **128**(2): 124–9.

7. Chirico V, Lacquaniti A, Salpietro V, Luca N, Ferraù V, Piraino B, et al. Thyroid dysfunction in thalassaemic patients: ferritin as a prognostic marker and combined iron chelators as an ideal therapy. *Eur J Endocrinol.* 2013; **169**(6): 785–93.

8. Ang AL, Tzoulis P, Prescott E, Davis BA, Barnard M, Shah FT. History of myocardial iron loading is a strong risk factor for diabetes mellitus and hypogonadism in adults with beta thalassemia major. *Eur J Haematol.* 2014; **92**(3): 229–36.

9. Chirico V, Rigoli L, Lacquaniti A, Salpietro V, Piraino B, Amorini M, et al. Endocrinopathies, metabolic disorders, and iron overload in major and intermedia thalassemia: serum ferritin as diagnostic and predictive marker associated with liver and cardiac T2 MRI assessment. *Eur J Haematol.* 2015; **94**(5): 404–12.

10. Poggi M, Sorrentino F, Pugliese P, Smacchia MP, Daniele C, Equitani F, et al. Longitudinal changes of endocrine and bone disease in adults with beta-thalassemia major receiving different iron chelators over 5 years. *Ann Hematol.* 2016; **95**(5): 757–63.
